# Loss of functional capacity in elderly individuals with Alzheimer disease

**DOI:** 10.1590/1980-57642020dn14-040009

**Published:** 2020-12

**Authors:** Susan Kelly Damião do Rego e Silva Andrade, Maria Clara Silva de Melo, Bartolomeu Fagundes de Lima, Fábio Henrique de Gobbi Porto, Vanessa Giffoni de Medeiros Nunes Pinheiro Peixoto, Juliana Maria Gazzola

**Affiliations:** 1 Universidade Federal do Rio Grande do Norte – Natal, RN, Brazil.; 2 Institute of Psychiatry, Universidade de São Paulo – São Paulo, SP, Brazil.

**Keywords:** Alzheimer disease, disability, executive function, postural balance, doença de Alzheimer, incapacidade, função executiva, equilíbrio postural

## Abstract

**Background:**

The functional capacity of elderly individuals with Alzheimer disease (AD) progressively declines.

**Objective:**

To verify the influence of sociodemographic, clinical, staging, mobility, and postural and cognitive balance data on the impairment of the functional capacity of elderly individuals with AD.

**Methods:**

This observational, analytical, cross-sectional study was performed at the Physiotherapy Department of the Universidade Federal do Rio Grande do Norte, Natal, Rio Grande do Norte, Brazil. The study consisted of forty elderly individuals aged ≥60 years old with mild or moderate AD, who could ambulate independently. The instruments used included a questionnaire to assess sociodemographic and anthropometric data; the Mini-Mental Health State Examination (MMSE); the Clinical Dementia Rating (CDR); a clock drawing test (CDT); a verbal fluency test (VFT); the Timed Up and Go Test (TUG); and the Clinical Test of Sensory Organization and Balance (CTSIB). Simple descriptive analyses, Mann-Whitney test, Spearman's correlation test, linear regression modeling, and prediction equation (p<0.05, 95% confidence interval [95%CI]) were performed.

**Results:**

Fifteen linear regression models were generated, with the final model chosen for analysis. The variables assumed in that model were CDR, MMSE score, and condition 3 of the CTSIB, which explained 60.1% of the outcome.

**Conclusions:**

Impairment of functional capacity in elderly individuals with AD was influenced by disease progression, which was due to cognitive deficits and deficits in postural balance, which are related to the inaccuracy of the somatosensory system in performing sensory integration.

## RESUMO.

**Introdução:**

A capacidade funcional de idosos com doença de Alzheimer (DA) sofrerá prejuízo progressivo.

**Objetivo:**

O presente estudo visou verificar a influências de dados sociodemográficos, clínicos, de estadiamento, mobilidade, equilíbrio postural e cognitivos no prejuízo da capacidade funcional de idosos com DA.

**Métodos:**

Trata-se de um estudo observacional, analítico e transversal, realizado no Departamento de Fisioterapia da Universidade Federal do Rio Grande do Norte, em Natal, Rio Grande do Norte, Brasil. O estudo contou com a participação de 40 idosos com idade igual ou superior a 60 anos com DA leve ou moderada, com deambulação independente. Os instrumentos utilizados incluíram um questionário para avaliação de dados sociodemográficos e antropométricos; o Mini-Exame de Estado de Saúde Mental (MEEM); a Avaliação Clínica da Demência (CDR); o Teste do desenho do Relógio (TDR); o Teste de Fluência Verbal (TFV); o Timed Up and Go Test (TUG); e o Clinical Test of Sensory Organization and Balance (CTSIB). Foram realizadas análises descritivas simples, teste de Mann-Whitney, teste de correlação de Spearman, modelo de regressão linear e equação de predição (p<0,05 e intervalo de 95% [IC95%]).

**Resultados:**

Foram gerados quinze modelos de regressão linear e foi escolhido o último para a análise. As variáveis assumidas nesse modelo citado foram: CDR, MEEM e condição três do CTSIB, que explicam 60,1% do desfecho.

**Conclusões:**

Pode-se concluir que o prejuízo da capacidade funcional de idosos com DA é influenciado pelo avançar da doença, pelos déficits cognitivo e de equilíbrio postural, sendo esse mais relacionado à imprecisão do sistema somatossensorial em realizar a integração sensorial.

## INTRODUCTION

Alzheimer disease (AD) is the most common neurodegenerative disease. It is irreversible and has a long and slow progression (mean of 8 to 12 years).^1^ ^,^ ^2^ AD has an estimated worldwide prevalence of 10–30% in the population aged >65 years.^3^ In Europe, the prevalence is reported to be to 5.5% (95% confidence interval [95%CI] 4.73–5.39%), with an incidence of 11.8 per 1,000 people/year (95%CI 10.30–11.89 people/year).^4^ The risk for onset is doubled every five years after 65 years of age, and, according to a systematic review from 2015, dementias in the Brazilian population feature a prevalence between 5.1 and 17.5%.^5^

After diagnosis of this condition, cognitive deficits emerge in the prodromal phase, characterized by mild cognitive deficits that become more evident, even interfering with the activities of daily living in the dementia stage.^6^ The first problems reported in everyday life typically involve more challenging activities, such as cooking, managing finances, and operating devices, which are generally referred to as instrumental activities of daily living (IADL) and are cognitively more complex.^7^ ^,^ ^8^ These progressive deficits in cognitive function(s) lead to impaired reasoning and planning, functional and social losses, as well as changes in behavior, with a consequent loss of functional independence.^9^ ^,^ ^10^

The Functional Activities Questionnaire (FAQ), developed by Pfefer,^11^ is the most used instrument for assessing IADL in Brazilian studies addressing the population with dementia.^12^ ^–^ ^14^ The instrument assesses performance in ten IADL. In addition, it can be used to distinguish individuals with cognitive loss from senility, from those with dementia through a better balance between sensitivity and specificity, when compared to the Lawton and Broody Scale.^11^ This instrument has been recently adapted to the Brazilian context.^14^

The aim of this study is to carry out an analysis of the impairment of the functional capacity of elderly people with AD in the face of a status, sociodemographic, clinical, mobility, postural, and cognitive balance factors.

## METHODS

### Sample population

The present investigation was an observational, analytical, cross-sectional study using a sample of individuals diagnosed with AD. Individuals were recruited at the Clinical Center of Ribeira in Natal, Rio Grande do Norte, Brazil, after undergoing geriatric evaluation, received the clinical diagnosis of probable Alzheimer disease, following the criteria of the Diagnostic and Statistical Manual of Mental Disorders (DSM–IV), of the Scientific Department of Cognitive Neurology and the Aging of the Brazilian Academy of Neurology.^15^ Disease staging was performed using the Clinical Dementia Rating Scale (CDR),^16^ ^,^ ^17^ in CDR 1 (light phase) or CDR 2 (moderate phase), and independent ambulation, or with the aid of a walking device.

Aged individuals with other neurological diseases, such as dementia from another etiology, Parkinson disease, and previous stroke, were excluded. Those who participated engaged in regular physical activity or physical therapy, and had the benefit of improving body balance in the previous six months. A total of 47 participants were contacted, 7 of whom were excluded due to visual and hearing complaints and severe cognitive impairment, thus leaving a total of 40 participants in the study, with 26 assessed as CDR 1 and 14 as CDR 2.

### Sample size calculation

The study sample was determined based on mean FAQ score for light level dementia (*i.e*., CDR1), which was the most prevalent. Thus, a mean FAQ score of 16 from a pilot study was used, with a tolerable absolute error of 5% and a presumed patient population of 5,000 subjects diagnosed with dementia, a 95%CI, and a test power of 80%. It was applied using the following equation:

n=(z_((1-γ)/2)^2 Nδ^2)/(d^2 (N-1)+z_((1−γ)/2)^2 δ^2)

This yielded a minimum sample size of 40 participants diagnosed with AD.

### Procedures and instruments

The present study was approved by the Research Ethics Committee of the Universidade Federal do Rio Grande do Norte, Brazil (No. 2.772.429). All participants, along with their family members/caregivers provided written informed consent for participation. In a routine consultation with the Geriatrics sector, as mentioned, potential participants were evaluated by the professional and, if the inclusion criteria were fulfilled, they were referred to participate in the study at a previously scheduled time.

Subjects and their companions received information about the objectives and basic research procedures. All subjects underwent individual evaluation in a suitable environment, which lasted for approximately 1 h 30 min. The evaluators were previously trained to provide security to the subjects. The evaluations were carried out from July 15^th^ to September 30^th^, 2018.

The analyzed variables were classified into the following categories: sociodemographic, dementia staging, anthropometric, cognitive, clinical, mobility, body balance, and functional capacity.

Sociodemographic data evaluated included gender, age (years), age range, annual income, education level, and years of schooling. Staging was assessed according to the CDR, which enables classification of the various degrees of dementia, assessing cognition and the influence of cognitive losses on the ability to properly perform activities of daily living. Anthropometric data included height, weight, and body mass index (BMI).^18^ The cut-off points used were: BMI≤22 kg/m^2^ (low weight); BMI≥22 kg/m^2^ (eutrophic); and BMI≥27 kg/m^2^ (overweight).^19^ The number of diseases, number of medications, and time of diagnosis of AD were also recorded.

Cognitive assessment was performed using the Mini-Mental Health State Examination (MMSE), a brief 30-point questionnaire used to track cognitive impairment. It is used to measure arithmetic function, memory, and orientation.^20^ ^,^ ^21^ The Clock Drawing Test (CDT) involves the task of drawing a clock with the insertion of hands at a designated time, in this case, 2 h 45 min. In this version, test scores range from 0 to 10, with higher scores indicating better performance.^22^ The Verbal Fluency Test (VFT) provides information about the storage capacity of the semantic memory system, the ability to retrieve information stored in memory, and the processing of executive functions, especially those through the ability to organize thinking and strategies used for word search.^23^

Clinical data evaluated included the number of diseases, type(s) of other diseases, number of medications, and types of medications used, which were reported by participants’ caregivers. To assess mobility, the volunteer was asked about the use of a walking aid device, the occurrence of falls in the previous six months, the fear of falls, and the presence of dizziness.

The Timed Up and Go test (TUG) consists of asking the subjects to rise from a chair, walk a distance of 3 m, turn around, and return. At the beginning of the test, subjects were instructed to support their spine on the back of the chair and return to this position at the end of the test. They received the instruction “go” to perform the test, and the time required from the moment the subject stood up to the moment they rested their back on the back of the chair was recorded. The test was performed once for familiarization and a second time for recording time.^24^ Three versions of the TUG were applied in the present study: conventional, in which the subject stood from a chair, walked 3 m, and returned to the same chair; sensitized, with a double motor task, in which the subject performs the activity carrying a glass filled with water in the dominant hand; and dual task (motor and cognitive), in which the subject performs the associated task while naming animals. In these tests, the time required to perform the tasks was recorded.^25^

The assessment of body balance was performed using the Sensory Interaction Test or Clinical Test of Sensory Organization and Balance (CTSIB). In this study, only four conditions were evaluated. In condition 1 (eyes open while on a firm surface), all senses are present. In condition 2 (eyes closed while on a firm surface), the visual system does not provide information. In condition 3 (eyes open while standing on foam surface), there is inaccurate information from the somato-sensitive system. Finally, in condition 4 (eyes closed while standing on foam surface), there is inaccurate information about the somato-sensitive system and absence of the visual system. A deficit in body balance was considered present when the subject exhibited displacement to one side, forward, or backward in at least one of the conditions.^26^ ^,^ ^27^

The assessment of functional capacity was performed using the FAQ, according to Pfeffer.^28^ The FAQ is a scale consisting of 10 questions applied to subjects’ companions or caregivers, addressing the ability of the subjects to perform certain functions. The responses followed a pattern: yes, he/she is capable (0); never did, but could do it now (0); with some difficulty, but he/she does it (1); never did and would have difficulty now (1); needs help (2); is not capable (3). A score ≥6 suggests greater dependence. The maximum score was 30 points. This questionnaire evaluates performance in 10 IADL that also involve cognitive skills, including managing one's own finances; shopping; heating water; putting out fire(s); preparing meals; keeping up to date; paying attention to the news and discussing it; remembering appointments; taking care of their own medication; maintaining orientation when walking around the neighborhood; and walking alone at home.^11^ ^,^ ^28^ ^,^ ^29^

### Statistical analyses

The FAQ quantitative variable according to Pfeffer was the study's dependent variable. A simple descriptive analysis (median, minimum, and maximum values) was calculated for all quantitative variables. Qualitative variables are expressed as absolute values and percentages, according to the characterization of the variable.

The Kolmogorov-Smirnov test was used to analyze the sample distribution, which was considered nonparametric. The Mann-Whitney test was used to relate the quantitative variable FAQ according to Pfeffer with the categorical variables. The correlation between the functional capacity quantitative variable and the other quantitative variables was assessed using the Spearman's correlation test.

The generalized linear model (GLM) was used. A gamma distribution with a ligand function Log was used, so that the independent variables could establish linear relationships with the outcome. Standardized (β) and non-standardized (B) regression coefficients were estimated. The β coefficient enabled comparison between independent variables. A significance level of 5% was adopted to minimize type I error in the adjustment of the model and independent variables.

## RESULTS

A total of 47 potentially eligible participants were contacted and 7 were excluded, thus leaving 40 subjects for final analysis, with 26 assessed as CDR 1 and 14 as CDR 2. Exclusion criteria included difficulty with telephone contact, presenting audiovisual complaints, and others for presenting cognitive complaints incompatible with the inclusion criteria.

Sociodemographic, anthropometric, clinical, cognitive, and mobility characteristics of the aged individuals with AD are summarized in Table 1. Data from the analysis of qualitative independent variables and the functional capacity are summarized in Table 2. Table 3 describes the associations between the independent quantitative variables and the functional capacity of elderly individuals with AD.

**Table 1:** Sociodemographic, anthropometric, clinical, cognitive, and mobility characterization of aged people with Alzheimer disease (n=40).

Characteristics (n=40)		n (%)	Median		Variation
Gender					
	Female	30 (75)			
	Male	10 (25)			
CDR					
	CDR1	26 (65)			
	CDR2	14 (35)			
Income					
	Up to 2 minimum wages	32 (80)			
	3 minimum wages or more	8 (20)			
Age			80.5	68–93	
Alzheimer disease diagnostic time (years)			2.00	1–10	
Functional capacity (Pfeffer)			21.00	6–28	
Age (full years)			80.50	68–93	
Schooling (years)			3.00	0–22	
MMSE			14.00	8–21	
BMI			25.10	14.9–40.4	
Clock design			2.00	1–10	
Verbal fluency			7.00	1–17	
Conventional TUG (seconds)			17.34	9.45–58.19	
TUG Dual Task – motor and cognitive (seconds)			24.05	14.20–92.0	
TUG Dual Motor Task (seconds)			18.70	11.48–80.0	

CDR: Clinical Dementia Rating; MMSE: Mini-Mental Health State Examination; BMI: body mass index; TUGT: Timed Up and Go Test; Pfeiffer: functionality test according to Pfeffer.

**Table 2:** Association between functional capacity and the variables of staging and postural balance of aged people with Alzheimer disease (n=40).

Qualitative independent variables and their categories		Functional capacity		p-value
		Median	Variation	
CDR	Light phase (26)	15.00	26–6	<0.001
	Moderate phase (14)	25.50	29–20	
Walking aid device	Uses (5)	17.00	25–14	0.854
	Not using (35)	20.00	29–6	
Falls in the last six months	Yes (16)	22.50	29–8	0.094
	No (24)	18.00	28–6	
Postural balance (CTSIB)	Good performance (18)	18.00	6–28	0.066
	Loss (22)	23.50	8–29	
Dizziness	Yes (24)	22.50	28–11	0.042
	No (16)	17.00	28–6	
Fear of falls	Yes (29)	22.00	29–8	0.785
	No (11)	16.00	28–6	

CDR: Clinical Dementia Rating; CTSIB: Clinical Test of Sensory Organization and Balance.

**Table 3:** Correlation between functional capacity and sociodemographic, anthropometric, clinical, cognitive, mobility, and postural balance variables of aged people with Alzheimer disease (n=40).

Quantitative independente variables	(rho)	p-value
MMSE	-0.57	<0.001
Age	0.26	0.097
Education	-0.20	0.195
Height	-0.27	0.086
Weight	-0.19	0.219
BMI	-0.06	0.704
Number of diseases	0.16	0.297
Number of medicines	-0.02	0.876
Alzheimer's diagnostic time	0.34	0.030
Clock design	-0.38	0.015
Verbal fluency	-0.59	<0.001
Conventional TUG (seconds)	0.42	0.007
TUG Dual Task – motor and cognitive (seconds)	0.53	<0.001
TUG Dual Motor Task (seconds)	0.44	0.005
CTSIB condition 1 (seconds)	0.27	0.083
CTSIB condition 2 (seconds)	0.36	0.020
CTSIB condition 3 (seconds)	0.32	0.039
CTSIB condition 4 (seconds)	0.31	0.050

rho: Spearman coefficient correlation or Spearman rô; MMSE: Mini-Mental Health State Examination; BMI: body mass index; TUG: Timed Up and Go Test; CTSIB: Clinical Test of Sensory Organization and Balance; Condition 1: eyes open on firm surface; condition 2: eyes closed on a firm surface; condition 3: eyes open on an unstable surface; and condition 4: eyes closed on an unstable surface.

A linear regression model was developed with the significant variables in the inferential analysis and those that obtained a p-value of up to 0.20, more specifically, CDR, falls in the previous 6 months, postural balance (CTSIB), dizziness, MMSE score, age, education, height, time of diagnosis of AD, CDT, VFT, conventional TUG (seconds), dual task — motor and cognitive TUG (seconds), dual motor task TUG (seconds), CTSIB condition 1 (seconds), CTSIB condition 2 (seconds), CTSIB condition 3 (seconds), and CTSIB condition 4 (seconds).

Fifteen linear regression models were generated, and the final model was adopted for the analysis using the “backward” method. The variables that remained until the final model included CDR, MMSE score, dizziness and condition 3 of the CTSIB (Table 4). Based on this analysis, these variables explained 61.1% of the decline in functional capacity in aged people with Alzheimer's.

**Table 4:** Multiple Linear Regression Based on the Functional Activities Questionnaire according to Pfeffer, in a sample of aged people with Alzheimer Disease.

Independent variables	Standard error	B	p-value
CDR	1.54	0.53	<0.001
MMSE	0.20	-0.27	0.021
Dizziness	1.38	-.17	0.097
Condition 3 of the CTSIB	0.57	-0.21	0.046

CDR: Clinical Dementia Rating; MMSE: Mini-Mental Health State Examination; CTSIB: Clinical Test of Sensory Organization and Balance; condition 3: eyes open on an unstable surface.

## DISCUSSION

After regression analysis, the variables that most influenced functional capacity were CDR, MMSE score, and condition 3 of the CTSIB, explaining 61.1% of the functional capacity outcome. The results of the CDR and MMSE were already expected in relation to the FAQ. Two variables, staging and cognitive performance, influenced the progress of the condition in this sample.^30^ ^–^ ^36^

As AD is a neurodegenerative condition, the significant relationship between MMSE and FAQ in this study (p<0.001) also agrees with studies reported in the literature, considering that the evolution of this condition is marked by the progression of cognitive deficit in these aged individuals. Sobral et al.^36^ reported a significant relationship between the MMSE score and CDR, which corroborates our results, confirming cognitive worsening with disease progression.

Assessing the functional capacity of individuals with Alzheimer disease can allow the professional to identify the severity of the motor impairment, as it may be associated with the stage of the disease, since cognitive and motor functions share neuroanatomical structures.^37^

The postural balance deficit in aged individuals with AD is reported to be an incidental event in this population, although uncommon, especially in the initial phase of the disease.^38^ ^–^ ^40^ Data from the present study corroborate those from previous ones,^38^ ^–^ ^41^ in which the progress of the disease was found to impair functional capacity and worsen the control of postural balance in more advanced stages of the disease.

The presence of balance deficit was not confirmed in relation to the association with the functional capacity of subjects in our sample, which confirms the findings reported in the literature.^41^ ^–^ ^43^ As most of our sample consisted of aged individuals with AD in the mild phase, in this case, the balance deficit still has a significant influence on the impairment of functional capacity.

The balance deficit was considered through displacement to one side, forward, or backward in at least one of the conditions of the CTSIB.^26^ ^,^ ^27^ Since it is a measure of static balance, this test is less influenced by the sample's cognitive biases, compared with tests such as the Berg Balance Scale (BBS), which requires greater complexity for its performance. Furthermore, the BBS has a ceiling effect for assessing postural balance, which can explain the good performance in this reported test.^39^ ^,^ ^41^

Condition 3 of the CTSIB evaluates the individual's body balance through a position in which the somato-sensitive system offers inaccurate information for the establishment of body balance.^26^ The fact that condition 3 of the CTSIB demonstrated significance in the final result of the regression analysis may suggest that the balance deficit in aged individuals with AD is related to the inaccuracy of somato-sensitive information for sensory integration in the central nervous system (CNS), which is necessary for postural control.

As the maintenance of posture and control of postural balance are influenced by the CNS and AD is a neurodegenerative process, impairment of the temporal and parietal cortex structures may be affected. The information from peripheral sensory receptors in the vestibular, visual, and somatosensory systems (somatosensation/proprioception) organized at the level of the CNS to provide motor actions appropriate to body balance^43^ ^,^ ^44^ can confirm the relationship between the functional capacity of elderly individuals with AD and postural balance deficit in condition 3 of the CTSIB (*i.e*., eyes open while standing on foam surface) as found in this study.

The relationship between suppression of the somatosensory system and postural balance in aged individuals with AD, although rare in the literature, can be understood with the help of studies such as the one by Cameron et al.,^45^ who, in a systematic review of the Cochrane meta-analysis library, investigated the use of transcutaneous electrical nerve stimulation (TENS) for dementia. The authors reported that TENS applied to the backs of patients with dementia can produce benefits by altering the activity of various neurotransmitters, inducing cholinergic, serotonergic, and noradrenergic changes. Furthermore, according to this meta-analysis, the somatosensory peripheral stimulus provided by TENS appeared to improve higher-level brain function more directly through ascending neural pathways that transmit information to the brain. The authors also cited animal studies that showed that peripheral somatic stimulation can cause CNS activation in several regions associated with memory functions, including the hippocampus, as well as the release of acetylcholine. In addition, patients subjected to TENS therapy in exploratory studies demonstrated a beneficial effect of TENS compared with placebo on delayed recall memory, facial recognition, and motivation assessed directly after treatment completion.^45^

These findings deepen the understanding of the influence of the somatosensory system in aged individuals with AD, although this information remains scarce in the literature. The understanding that the somatosensory system is one of the bases of sensory integration necessary for body balance and the fact that the stimuli source of somatosensory receptors are capable of promoting activation of neurotransmitters, such as acetylcholine, may explain why the elderly in this sample were not able to maintain body balance during suppression of somato-sensitive information.^44^ ^,^ ^46^

Corroborating with our data, a cross-sectional study recently published by Yoon et al. points out the relationship between cognitive decline and balance, when assessing balance through TUG and unipodal support and cognitive deficit through the Montreal Cognitive Assessment (MOCA) scale, in 295 participants, among aged controls without cognitive deficit and aged people with different levels of cognitive deficit, even with AD. These authors pointed out that the altered neural network or amyloid or tau deposition that have already been triggered may have affected the balance function.^47^

Since functional capacity is an important characteristic in AD, this relationship points to the postural balance of aged individuals with AD, an influential condition in the progression of the disease. These data should guide the professional practice of physical therapists and other health professionals to resolve functional damage.

The limitations of this study refer to the difficulty of transporting these patients, since the presence of a caregiver is necessary, as the aged already have some mobility difficulties, as well as to the fact that this is related to the same medical service, which may have tended to homogenize the sample in relation to clinical data, and the design of the study that only allows correlation analyses, which do not allow inferring the cause and effect relationship; for that, it is suggested that future studies perform longitudinal analyses.

It can be concluded that the functional capacity of elderly people with AD may be related to the progress of the disease, cognitive deficit, and postural balance deficit, which is more related to the accuracy of the somatosensory system in performing sensory integration. And these characteristics can have a great influence on the autonomy of this population, more markedly, with the progress of the condition.
